# Food Labels Use Is Associated with Higher Adherence to Mediterranean Diet: Results from the Moli-Sani Study

**DOI:** 10.3390/nu5114364

**Published:** 2013-11-04

**Authors:** Americo E. Bonanni, Marialaura Bonaccio, Augusto di Castelnuovo, Francesca de Lucia, Simona Costanzo, Mariarosaria Persichillo, Francesco Zito, Maria Benedetta Donati, Giovanni de Gaetano, Licia Iacoviello

**Affiliations:** 1 Epicomed Research Srl, Campobasso 86100, Italy; E-Mail: bonanni@filemazio.net; 2 Department of Epidemiology and Prevention, IRCCS Istituto Neurologico Medieterraneo Neuromed, Pozzilli 86077, Italy; E-Mails: marialaura.bonaccio@moli-sani.org (M.B.); dicastel@ngi.it (A.C.); simona.costanzo@moli-sani.org (S.C.); mariarosaria.persichillo@moli-sani.org (M.P.); mbdonati@moli-sani.org (M.B.D.); giovanni.degaetano@moli-sani.org (G.G.); 3 Cuoresano Onlus, Campobasso 86100, Italy; E-Mail: fdelucia202@gmail.com; 4 U.O.C. Medicina Trasfusionale e Immunoematologia, Ospedale Veneziale di Isernia, Isernia 86170, Italy; E-Mail: francesco.zito@moli-sani.org

**Keywords:** Mediterranean diet, food labels, dietary patterns, cardiovascular diseases, chronic diseases

## Abstract

Mediterranean diet (MD) has been associated with lower risk of ischemic cerebro- and cardio-vascular disease, neurological degenerative disease, and breast and colonrectal cancers. Nevertheless, adherence to this pattern has decreased. Food labels are a potentially valid means to encourage towards healthier dietary behavior. This study, conducted on a subsample of 883 subjects enrolled in the Moli-sani Project, evaluated whether food labels reading (LR) is associated with MD adherence. Participants completed a questionnaire on nutrition knowledge, information, and attitudes, with a specific question on food labels reading. Biometric measurements, socio-economic status, education, physical activity, and smoking habits were collected. The European Prospective Investigation into Cancer and Nutrition (EPIC) food frequency questionnaire was used to collect dietary habits, and subsequently evaluated by both the Mediterranean diet score (MDS) and Italian Mediterranean index (IMI), *a priori* dietary patterns. Food consumption patterns were generated by Principal Components Analysis (PCA), an *a posteriori* approach. Multivariable odds ratios were calculated to quantify the association of LR categories with dietary habits. LR was significantly associated with greater adherence to both MDS (*p* = 0.0004) and IMI (*p* = 0.0019) in a multivariable model. LR participants had 74% (MDS) or 68% (IMI) higher probability to be in the highest level of adherence to Mediterranean diet-like patterns. Moreover, they showed greater adherence to Mediterranean-like food consumption patterns (0.1 *vs*. −0.2, *p* < 0.0001) and lower adherence to two Western-like patterns (0.01 *vs*. 0.2, *p* = 0.009 and 0.1 *vs*. 0.2, *p* = 0.02). These findings support an association between food label use and consuming a Mediterranean-type diet.

## 1. Introduction

Mediterranean diet (MD) has been associated with lower incidence of chronic degenerative diseases such as cardiovascular disease, stroke, neurodegenerative diseases, and some forms of cancer [1,2,3,4]. Moreover, evidence of a protective effect of MD on cardiovascular and neurovascular diseases has been provided by recent intervention studies [5]. In the wake of these data, the Mediterranean diet has been proposed as an optimal strategy of public health intervention [6].

However, in spite of the growing scientific and cultural [7] support, MD is being abandoned in the areas from which it originated, such as in Southern European and Northern African countries [8,9]. The adherence to this dietary pattern is, particularly, decreasing among young people in countries with strong Mediterranean traditions [10,11], foreseeing a deteriorating situation in future adults, probably also favored by economic factors. Indeed, low income is associated with lower adherence to MD-like eating patterns [12]. In this context, examination of approaches to improve MD adherence, especially among those population historically linked to Mediterranean tradition, is warranted.

Nutrition information on food labels, that have been standardized in the USA by the 1990 Nutrition Labeling and Education Act [13], and made mandatory by the European Commission in December 2011 [14], is regarded as a potentially valid mean to encourage people towards generally healthier dietary behaviors [15]. Research show that label use is effectively associated with the consumption of healthier nutrients [16,17] and, in particular, it can have a positive effect on choosing beneficial nutrient components and a negative effect on harmful ones [18].

To our knowledge, no study addressed the impact that food labels could have on the adherence to complex dietary patterns. Therefore, the aim of the present study was to evaluate the association between labels reading and adherence to the Mediterranean diet in an adult population living in a central-southern region of Italy, with strong MD traditions.

## 2. Methods

### 2.1. Study Population

The study includes a subsample of subjects enrolled in the Moli-sani Project, a population-based cohort study on citizens resident in the Molise region, placed between Central and Southern Italy. The Moli-sani study started in 2005 and randomly recruited men and women aged ≥35 years from subjects included in the city-hall registries of Molise [19]. Exclusion criteria were pregnancy, disturbances in understanding/willing processes, ongoing poly-traumas or coma, refusal to sign the informed consent; 30% of subjects refused to participate; these were generally older and had a higher prevalence of cardiovascular disease. At the end of recruitment, in April 2010, 24,325 subjects were enrolled. In 2009, we elaborated an additional self-administered questionnaire on nutrition knowledge and mass media exposure. This questionnaire was proposed to participants, recruited from May 2009 to April 2010 (*n* = 1571). The rate of participation to the nutrition knowledge and mass media questionnaire was 61%. After exclusion of 75 questionnaires with missing data for relevant questions, the final sample size was 883 subjects.

The Moli-sani study was approved by the Ethics Committee of the Catholic University of Rome. All participants signed the informed consent before taking part in the study.

### 2.2. Dietary Information

The Italian validated European Prospective Investigation into Cancer and Nutrition (EPIC) food frequency questionnaire (FFQ) was administered in-person to determine food intake [20,21]. The questionnaire, computerized with tailor-made software, allowed to interview participants in an interactive way, including illustrations of sample dishes of definite sizes or by reference to standard portion sizes. To simplify interpretation of data and to minimize within-person variations in intakes of individual foods, 188 food items were classified into 45 predefined food groups on the basis of similar nutrient characteristics or culinary usage.

### 2.3. Dietary Analysis

To obtain a broader picture of healthy habits, we evaluated the adherence to a Mediterranean diet using two scores. The first one was the Mediterranean diet score (MDS), elaborated by Trichopoulou *et al*. [22], whereas the Italian Mediterranean index (IMI) [23], was specifically elaborated to better capture healthy eating in Italy, including foods, such as pasta, more typically consumed in this country.

MDS scoring was based on the intake of the following nine items: vegetables, legumes, fruits and nuts, dairy products, cereals, meat and meat products, fish, alcohol, and the ratio of monounsaturated-saturated fats. For most items, consumption above the study median received 1 point; all other intakes received 0 points. For dairy products and meat, of which consumption is considered unhealthy, an intake below the median received 1 point. Medians are gender specific. For ethanol, men who consumed 10–50 g/day and women who consumed 5–25 g/day received 1 point; otherwise, the score was 0. The possible scores ranged between 0 and 9, the latter value reflecting the maximal adherence.

IMI scoring was based on the intake of 11 items: a high intake of six typical Mediterranean foods (pasta; typical Mediterranean vegetables such as raw tomatoes, leafy vegetables, onions, and garlic, salads, and fruiting vegetables; fruit; legumes; olive oil; and fish); a low intake of four non-Mediterranean foods (soft drinks, butter, red meat, and potatoes); and alcohol consumption. If the consumption of typical Mediterranean foods was in the third tertile of the distribution, the participant received 1 point; all other intakes received 0 points. If the consumption of non-Mediterranean foods was in the first tertile of the distribution, the participant received 1 point. Ethanol received 1 point for intake up to 12 g/day; abstainers and participants who consumed >12 g/day received 0 points. Possible scores ranged from 0 to 11. High adherence to MD, as stated by MDS, was defined when the score was >4 points, whereas low adherence when the score was <4 points. For IMI, respectively, high and low adherence were at >3 and <3 points. Subjects with intermediate values (MDS = 4 or IMI = 3) were excluded from this analysis to focus on the two extreme categories of adherence to MD.

The food consumption pattern was obtained using principal component analysis (PCA). The purpose of PCA is to derive a small number of components that can account for the variability found in a relatively large number of measures. This procedure, called data reduction, is typically performed when a researcher does not want to include all of the original measures in analyses, but still wants to work with the information that they contain. PCA was conducted on the correlation matrix of 45 food groups [24]. In determining the number of factors to retain in PCA we used the criterions of an eigenvalue >1.0, the scree test and the interpretability of the final solution [25]. Three main factors emerged with PCA, in agreement with previous findings in the same population [24]. The first pattern, named “Olive Oil and Vegetables”, was characterized by high positive loadings of olive oil, vegetables, legumes, soups, fruits, and fish. The second pattern, named “Pasta and Meat”, was characterized by high positive loadings of pasta, cooked tomatoes, red meat, animal fats, and alcoholic beverages, and negative loadings of breakfast cereals, and yogurt. The “Eggs and Sweets” pattern was characterized by high positive loadings of eggs, margarines, processed meat, sugar, and sweets. Food grouping used in the dietary pattern analyses are shown in Table 1.

**Table 1:** Food grouping used in the dietary pattern analyses.

Foods or Food Groups	Food Items
Potatoes	Potatoes
Cooked vegetables	Leafy vegetables, root vegetables, cabbages, onion, carrots, mushrooms, egg plants, artichokes, sweet peppers, spinach, pumpkins, canned vegetables in oil, picked vegetables
Raw vegetables	Raw leafy vegetables, raw tomatoes
Tomatoes (cooked)	Tomato sauces, tomatoes
Legumes	Beans, lentils, peas, chick peas
Fruit	Apples, pears, kiwi, bananas, grapes, peaches, apricots, oranges, tangerines, plums, strawberries, melon, khaki, figs, cherries
Nuts and dried fruit	Peanuts, almonds, hazelnuts, walnuts, dried figs, dried dates, prune
Olives	Olives
Milk	Milk
Yogurt	Yogurt
Fresh cheese	Mozzarella, ricotta cheese, taleggio cheese, gorgonzola cheese, melted cheese slices, other soft cream cheese
Seasoned cheese	Fontina cheese, emmenthal, gruyere, parmesan, caciocavallo cheese, other seasoned cheese
Pasta and other grains	Pasta, yellow maize meal
Rice	Rice
Bread	White bread, bread with oil, and other bread
Crisp bread, rusks	Breads sticks, crisp bread
Breakfast cereals	Breakfast cereals
Salty biscuits	Crackers
Red meat	Beef, pork, lamb, horse, game, veal, other meats
White meat	Chicken, turkey, rabbit
Processed meat	Sausages, ham, bologna sausage, dried beef, salami
Offals	Liver, offals
Canned fish	Canned tuna fish and other fish
Crustaceans, molluscs	Crustaceans, molluscs
Fish	Other fish
Egg	Eggs
Vegetables oils	Seed oils (except olive oils)
Olive oil	Olive oil
Butter	Butter
Margarines	Margarines
Animal fats	Visible fat from meat, poultry skin, fat from ham
Sugar & sweets	Sugar, honey, cakes, ice cream, confections, pastry, pudding
Fruit juices	Orange juice, grapefruit juices, other fruit juices
Soft drinks	Soft drinks
Coffee	Coffee
Tea	Tea
Other sauces	Dressing sauces for pasta other than tomato sauce
Mayonnaises	Mayonnaises
Soups	Vegetable soups
Bouillon	Meat and stock-cube broth
Snacks	Vegetable quiche
Pizza	Pizza
Wine	Red wine, rosé wine, white wine
Spirits	Alcoholic beverages other than wine or beer
Beer	Beer

### 2.4. Data Collection

Body mass index (BMI) was calculated as kg/m^2^. Lifestyles were assessed by a structured questionnaire including smoking habit and physical activity. Regarding the latter, 24 questions were asked on working time, leisure time, and sport participation. Physical activity was expressed as daily energy expenditure in metabolic equivalent task-hours (MET/day) [26].

### 2.5. Socioeconomic Variables

Household net income categories were considered as low (<25,000 euro/year), medium (>25,000 and ≤40,000 euro/year), and high (>40,000 euro/year).

Education level was considered as low (≤8 years) or high (>8 years). Socioeconomic status (SES) was expressed as a score based on five variables: dwelling ownership and ratio between the number of rooms and number of living-in family members (rooms per person), both currently and during childhood, and availability of hot water at home during childhood. The five components were dichotomized according to the median value, and a score of one was attributed to the category supposed to be marker of higher social status in comparison with the opposite category: thus we assigned a score of 1 to people living in a house with living-in family members/room density >0.6 or dwelling ownership or with availability of hot water, and a score 0 to people with living-in family members/room density ≤0.6, no dwelling ownership or with unavailability of hot water. We recorded missing values for SES for 5.7% of the subjects.

### 2.6. Food Labels Reading

The instrument for measuring food labels use was included in a larger questionnaire aimed at studying exposure to media information, trust in various health information sources, health, and dietary knowledge and attitude toward healthy behavior (the “Moli-info” questionnaire, developed by some of us (AB, MB, LI) and available upon request) [27,28]. A specific question was devoted to food labels reading (Table 2). To establish validity, test-retest method was used by administering twice, with a two-weeks separation, the information, knowledge, and attitude questionnaire to a group (*n* = 28) of researchers at the Fondazione di Ricerca e Cura “Giovanni Paolo II” of Campobasso.

**Table 2:** Attention to food labels’ single items.

Items	*n*	%
Usually, which item do you look at in food labels?		
Calories	194	22
Proteins	28	3.2
Carbohydrates	15	1.7
Fats	227	25.7
Fibers	12	1.4
Sodium	20	2.3
**Total (use labels)**	**496**	**56.2**
Usually I do not read food labels	268	30.2
Don’t know	119	13.5
**Total (do not use labels)**	**387**	**43.8**

Consecutive subjects (*n* = 1132) recruited in the Moli-sani study from May 2009 to April 2010 were asked to fill the self-administered questionnaire; participants reporting missing values were excluded.

People declaring to read any of the food labels’ items were classified as readers while those answering “usually I do not read food labels” or “do not know” constituted the non-reader group.

### 2.7. Validation

Taken as a whole, the Moli-info questionnaire have been validated for a Nutrition knowledge score and a Mass media exposure score. Scores between test and retest groups were highly correlated (Sperman correlation coefficient *r* = 0.71, *P* < 0.0001 for knowledge and *r* = 0.84, *P* < 0.0001 for Mass media exposure). The consistency with which participants answered the single question regarding label reading was assessed using Cohen’s Kappa, showing substantial consistency (Kappa = 0.63).

### 2.8. Statistical Analysis

Values for continuous variables are means ± Standard Deviation.

In validation, scores between test and retest groups were evaluated by using Spearman correlation. Consistency with which participants answered the single question regarding label reading was assessed using Cohen’s Kappa.

Multivariable analysis of variance for continuous or categorical variables was used for testing the associations of general characteristics or adherence to Mediterranean diet scores or other dietary pattern (considered as the dependent variables) with LR categories. The potential predictors tested for association with labels reading included socio-demographic variables (age, sex, income, smoking, educational level, and socioeconomic status), BMI, total caloric intake, smoking habit, and physical activity (expressed as MET/day). Basic model included age and sex, whereas fully adjusted model included age, sex, total caloric intake, socio-economic status, income, BMI, smoking, physical activity, and educational level. By using multivariable logistic regression analysis, odds ratio (ORs) with corresponding 95% confidence intervals (95% CI) were calculated to quantify the association of LR categories with food consumption patterns.

A specific category “non-respondent” was created for income and SES in order to consider the missing values in the multivariable analyses.

The data analysis was performed using SAS/STAT software [29].

## 3. Results

### 3.1. Study Sample

The final study sample was 883 subjects. Subjects who were excluded from the analysis because of missing relevant data (*n* = 75) were comparable for sex distribution, but were older (52.5 ± 9.5 *vs*. 58.5 ± 11.3; *p* value < 0.0001), and had lower social status (6.40 ± 2.34 *vs*. 7.42 ± 2.35; *p* value < 0.0001) in comparison with subjects included. It is likely that the whole questionnaire was too articulated to be completed by older participants and those having lower social status because they might find it, largely addressing modern technologies, too far away from their daily experience, and refrained from completing it properly. Yet the 883 analyzed subjects were comparable with the whole Moli-sani Project population in terms of sex distribution, cardiovascular disease, and metabolic syndrome prevalence, BMI, cardiovascular risk and dietary patterns (all *p* > 0.05), whereas mean age was lower (52.5 ± 10 *vs*. 55.6 ± 11.9, *p* < 0.0001).

### 3.2. Population Characteristics and Label Reading

Table 3 shows characteristics of the sub-sample of the Moli-sani project included in this study. 56.2% of participants reported to consult information in food labels.

**Table 3:** Characteristics of the population sample.

Characteristics		Read Food Labels		*P*-Value	
	All *n* = 883	No *n* = 387 (43.8%)	Yes *n* = 496 (56.2%)	Univariate	Sex/Age Adjusted
Age (year)	52.5 (9.6)	53.3 (9.3)	51.9 (9.2)	0.03	0.05
Gender				0.0004	0.0006
Women *n*, %	441 (49.9)	167 (43.2)	274 (55.2)		
Men *n*, %	442 (50.1)	220 (56.8)	222 (44.8)		
Smokers *n*, %				0.2	0.7
Never	370 (41.9)	150 (38.8)	220 (44.4)		
Current	243 (27.5)	109 (28.2)	134 (27)		
Former	270 (30.6)	128 (33.7)	142 (28.6)		
BMI	27.9 (4.6)	28.0 (4.4)	27.8 (4.8)	0.6	0.9
Total caloric intake (kcal)	2089 (607)	2114 (622)	2069 (596)	0.3	0.6
Physical activity (MET-h/day)	43.8 (8.2)	43.3 (8.1)	44.1 (8.3)	0.2	0.2
Social status score *n*,%				0.7	0.7
Low	221 (25)	104 (26.9)	117 (23.6)		
Medium	258 (29.2)	110 (28.4)	148 (29.8)		
High	354 (40.1)	153 (39.5)	201 (40.5)		
Non respondent	50 (5.7)	20 (5.2)	30 (6.1)		
Education level *n*, %				0.4	0.5
Low (≤8 years of education)	425 (48.2)	192 (49.7)	233 (47)		
High (>8 years of education)	457 (51.8)	194 (50.3)	263 (53)		
Income *n*,%				0.8	0.6
Low	359 (40.7)	154 (39.7)	205 (41.3)		
Medium	196 (22.2)	83 (21.5)	113 (22.8)		
High	64 (7.2)	29 (7.5)	35 (7.1)		
Non respondent	264 (29.9)	121 (31.3)	143 (28.8)		

Both in univariate and in multivariable (adjusted for age and sex) analysis, the label reading attitude was significantly more diffused among women than men. People using food labels information were slightly younger, an observation significant in univariate analysis and borderline significant in multivariable. No significant associations were found for smoking habits, BMI, total caloric intake, physical activity, social status score, education level, and income.

### 3.3. Association between Label Reading and Dietary Patterns

People reading food labels information showed a higher adherence to Mediterranean diet according both to MDS and IMI (Table 4). This association was observed in the basic model (*p* = 0.0005 MDS, *p* = 0.002 IMI), as well as in the fully adjusted model (*p* = 0.0004 MDS, *p* = 0.0019 IMI).

**Table 4:** Differences in adherence to Mediterranean diet and in dietary patterns between food labels readers and non-readers.

Mediterranean Diet and Dietary Patterns	Read Food Labels Mean ± Standard Deviation		*P*-Value	
	No *n* = 387	Yes *n* = 496	Basic Model	Fully Adjusted Model
Mediterranean Diet Score (MDS)	4.2 (1.6)	4.5 (1.6)	0.0005	0.0004
Italian Mediterranean Index (IMI)	3 (1.5)	3.3 (1.6)	0.002	0.0019
Dietary Pattern 1 (Olive Oil and Vegetables)	−0.2 (0.8)	0.1 (0.8)	<0.0001	<0.0001
Dietary Pattern 2 (Pasta and Meat)	0.2 (0.8)	0.01 (0.9)	0.002	0.009
Dietary Pattern 3 (Eggs and Sweets)	0.2 (0.9)	0.1 (0.8)	0.03	0.02

Basic model: Adjusted for age and sex; Fully adjusted model: Adjusted for age, sex, total caloric intake, socio-economic status, income, body mass index (BMI), smoking, physical activity, and educational level.

Regarding food consumption patterns, the label reading group showed a higher adherence to the pattern “Olive oil and vegetables” than non-readers, both in the sex/age adjusted model (*p* < 0.0001) and in the fully adjusted model (*p* < 0.0001). Readers group had a lower adherence to both patterns “Pasta and meat” and “Eggs and sweets” in multivariable model (respectively *p* = 0.009 and *p* = 0.02).

Such findings remains unchanged when we considered specific components of the food label. In particular, we did not find difference when only subjects declaring to pay attention to calories (*n* = 194) or fats (*n* = 227) or other items (*n* = 75) were compared to non-readers (data not shown).

MDS was then divided into two categories to get both a minimum (score from 0 to 3) and a maximum adherence (score > 4). The same was done with IMI score, obtaining a minimum from 0 to 2 and a maximum > 3.

In multivariable logistic regression model controlling for age, sex, total caloric intake, socio-economic status, income, BMI, smoking, physical activity, and educational level, odds of eating Mediterranean were higher in the label reader group than in non-readers. In fact, participants reading food labels had 73% higher probability to have greater adherence to MDS (OR 1.73, 95% CI: 1.24–2.42) (Table 5), and 67% to IMI (OR 1.67, 95% CI: 1.2–2.33) (Table 6).

**Table 5:** Odds ratios of having high adherence to Mediterranean diet score (MDS) according to food labels reading.

	Adherence to MDS					
Read Food Labels	Low (<4)	High (>4)	Model 1		Model 2	
	*n* = 265	*n* = 413	OR	(95% CI)	OR	(95% CI)
No	138 (52.1%)	167 (40.4%)	−1	-	−1	-
Yes	127 (47.9%)	246 (59.6%)	1.69	(1.23–2.33)	1.73	1.24–2.42

**Table 6:** Odds ratios of having high adherence to Italian Mediterranean index (IMI) according to food labels reading.

	Adherence to IMI					
Read Food Labels	Low (<3)	High (>3)	Model 1		Model 2	
	*n* = 310	*n* = 348	OR	(95% CI)	OR	(95% CI)
No	152 (49%)	132 (37.9%)	−1	-	−1	-
Yes	158 (51%)	216 (62.1%)	1.57	1.14–2.14	1.67	1.2–2.33

## 4. Discussion

In our study, 56.2% of participants reported to consult information on food labels at least for one item. This percentage is similar to the one found in a USA study (61.5%) [16], albeit ample variability has been found by previous researches. In the UK, food label reading was found to be 27% [30], while in other studies conducted in Europe it ranged from 18% to 63% [31]. This discrepancy could be accounted by cultural and socio-economic difference among populations included in the studies. According to the prevalence of reading observed in our study, food labels may represent one of the means by which a typical Mediterranean population receive information that, arguably, can be part of the decision process about food consumption in the same way it has been observed in other geographical areas [15,16,17,18].

In this context, we found that food labels consulting is associated with greater adherence to Mediterranean diet according to two different scores, MDS [22] and IMI [23]. These findings contribute to provide evidence that food labels are associated not merely to single healthy-oriented item choices, as stated by other studies [15,16,17,18], but also to a whole, complex dietary pattern. This is corroborated by odds-ratio analysis showing higher probability for label readers to be in the highest level of MD adherence, again both for MDS and for IMI.

To further evaluate the association between label reading and dietary patterns, beside an “*a priori*” scales like MDS and IMI, we used PCA, which is able to empirically derive dietary patterns not set *a priori*. This analysis showed that in our population food labels reading is positively associated with a healthy food pattern, close to the Mediterranean diet (“Olive oil and vegetables”) and negatively with other, less healthy, patterns (“pasta and meat”, “eggs and sugar”).

These observations suggest that people paying attention to food labels have a nutrition behavior, not only healthier, but closer to the Mediterranean model, albeit with an observed limited difference of just 0.3 points both for MDS and IMI. A limited amount of variance in nutrients intake explained by food labels reading have been reported also in other studies [16,32], but the association could still have sufficient magnitude to have potential impact [16].

In our findings the only socio-demographic characteristics having effect on food label reading are gender and, marginally, age, unlike other studies in which also education level and income do have an effect [16,18]. Social and cultural difference between Italian and other populations included in the studies could be a possible explanation for the discrepancy.

### Limitations of the Study

The first limitation in this study is its cross-sectional nature. Nevertheless, this type of investigation is useful to set new hypotheses to be tested in future prospective studies. Secondly, caution is needed in extending the results presented here to larger contexts as data were collected in a region located between Central and Southern Italy, Mediterranean by tradition and culture [19]. However, the main characteristics of our sample are comparable to those of the Italian Cardiovascular Epidemiological Observatory [33], a large survey including random samples of the general population all over Italy; therefore our sample could be considered representative, at least, of the Italian population.

Another weak point is the possibility of over-reporting of food label reading, due to social desirability bias, as suggested by previous findings [34]. We think we partially overcame this issue with the specific question asking not if participants read labels in general, but inquiring about which item they consult more often.

Finally, we must consider that the observed association between labels reading and Mediterranean diet adherence does not prove that labels use by consumers has impact on dietary habits. This much-debated issue was not addressed by the present study. Changing decisions in buying or not a specific product after reading labels have been previously reported for one-third of consumers [35], but it must be considered that a recent study observed no modification toward healthy choices in supermarket sales after introduction of “traffic light” labels [36].

## 5. Conclusions

This study highlights, to our best knowledge for the first time, the association between food labeling and healthy nutrition choices, not just in terms of single food or single nutrients, but also in the wider range of a complete dietary pattern. We cannot exclude that this association may only reflect a healthy lifestyle in general, and the potential role of food labeling in encouraging healthy dietary habits cannot be concluded without further research. More evidence in this direction could lead to a new concept of labeling, aimed to present foods according to whether they belong to the Mediterranean diet, and to what extent. This could be used in the framework of already used, new informative messages (Front-of-Package systems), such as the “traffic light” system developed by the United Kingdom Food Standards Agency [37]. Our findings should address specific public health campaign to implement food label use and reading to maintain Mediterranean dietary habits and the linked health advantages.

## Appendix

### Moli-Sani Project Investigators

**Chairperson**: Licia Iacoviello (Campobasso, Italy).

**Steering Committee**: Maria Benedetta Donati (Campobasso, Italy) and Giovanni de Gaetano (Campobasso, Italy) (Chairpersons), Simona Giampaoli (Roma, Italy).

**Safety and data monitoring Committee**: Jos Vermylen (Leuven, Belgio) (Chairman) Ignacio De Paula Carrasco (Roma, Italy), Enrico Garaci (Roma, Italy).

**Event adjudicating Committee**: Deodato Assanelli (Brescia, Italy), Francesco Alessandrini (Campobasso, Italy), Vincenzo Centritto (Campobasso, Italy), Paola Muti (Roma, Italy), Holger Schünemann (Hamilton, Canada), Pasquale Spagnuolo (Termoli, Italy), Dante Staniscia (Termoli, Italy), Sergio Storti (Campobasso, Italy).

**Scientific and organizing secretariat**: Francesco Zito (Coordinator, Campobasso and Termoli, Italy), Americo Bonanni (Campobasso, Italy), Chiara Cerletti (Campobasso, Italy), Amalia De Curtis (Campobasso, Italy), Augusto Di Castelnuovo (Campobasso, Italy), Licia Iacoviello (Campobasso, Italy), Antonio Mascioli (Campobasso, Italy), Marco Olivieri (Campobasso, Italy).

**Data management and analysis** (Campobasso, Italy): Augusto Di Castelnuovo (Coordinator), Antonella Arcari, Floriana Centritto (till December 2008), Simona Costanzo, Romina di Giuseppe, Francesco Gianfagna.

**Informatics** (Campobasso, Italy): Marco Olivieri (Coordinator), Maurizio Giacci, Antonella Padulo (till September 2008), Dario Petraroia (till September 2007).

**Biobank and biochemical analyses** (Campobasso and Termoli, Italy): Amalia De Curtis (Coordinator), Sara Magnacca, Federico Marracino (till June 2009), Maria Spinelli, Christian Silvestri (till December 2007), Cristina Vallese (till September 2008).

**Genetics** (Campobasso, Italy): Daniela Cugino, Monica de Gaetano (till October 2008), Mirella Graziano, Iolanda Santimone, Maria Carmela Latella (till December 2008), Gianni Quacquaruccio (till December 2007).

**Communication** (Campobasso, Italy): Americo Bonanni (Coordinator), Marialaura Bonaccio, Francesca De Lucia.

**Moli-family Project** (Campobasso, Italy): Branislav Vohnout (Coordinator) (till December 2008), Francesco Gianfagna, Andrea Havranova (till July 2008), Antonella Cutrone (till October 2007).

**Recruitment staff** (Campobasso and Termoli, Italy): Franco Zito (General Coordinator); Secretariat: Mariarosaria Persichillo (Coordinator), Angelita Verna, Maura Di Lillo (till March 2009), Irene Di Stefano (till March 2008); Blood sample: Agostino Pannichella, Antonio Rinaldo Vizzarri, Branislav Vohnout (till December 2008), Agnieszka Pampuch (till August 2007); Spirometry: Antonella Arcari (Coordinator), Daniela Barbato (till July 2009), Francesca Bracone, Simona Costanzo, Carmine Di Giorgio (till September 2008), Sara Magnacca, Simona Panebianco (till December 2008), Antonello Chiovitti (till March 2008), Federico Marracino (till December 2007), Sergio Caccamo (till August 2006), Vanesa Caruso (till May 2006); Electrocardiogram: Livia Rago (Coordinator), Daniela Cugino, Francesco Zito, Alessandra Ferri (till October 2008), Concetta Castaldi (till September 2008), Marcella Mignogna (till September 2008), Tomasz Guszcz (till January 2007); Questionnaires: Romina di Giuseppe, (Coordinator), Paola Barisciano, Lorena Buonaccorsi, Floriana Centritto (till December 2008), Francesca De Lucia, Francesca Fanelli (till January 2009), Iolanda Santimone, Anna Sciarretta, Maura Di Lillo (till March 2009), Isabella Sorella (till September 2008), Irene Di Stefano (till March 2008), Emanuela Plescia (till December 2007), Alessandra Molinaro (till December 2006), Christiana Cavone (till September 2005).

**Call Center** (Campobasso, Italy): Giovanna Galuppo (till June 2009), Maura Di Lillo (till March 2009), Concetta Castaldi (till September 2008), Dolores D’Angelo (till May 2008), Rosanna Ramacciato (till May 2008).
